# Contemporary management of drug-packers

**DOI:** 10.1186/1749-7922-2-9

**Published:** 2007-04-20

**Authors:** J Kelly, M Corrigan, RA Cahill, HP Redmond

**Affiliations:** 1 Department of Surgery, Cork University Hospital, Wilton, Cork, Ireland

## Abstract

Experience with management of drug-packers (mules) is variable among different centres. However, despite a recorded increase in drug trafficking in general, as yet, no unified, clear guidelines exist to guide the medical management of those who only occasionally encounter these individuals. We describe our recent experience with this growing problem and discuss the most salient points concerning the contemporary management of body packers. Our recent experience demonstrates that type IV packages may now be managed conservatively for the most part.

## Background

Experience with management of drug-packers ("mules") is variable among different centres. However, despite a recorded increase in drug trafficking in general, as yet, no unified, clear guidelines exist to guide the medical management of those who only occasionally encounter these individuals.

## Case report

**Figure 1:**
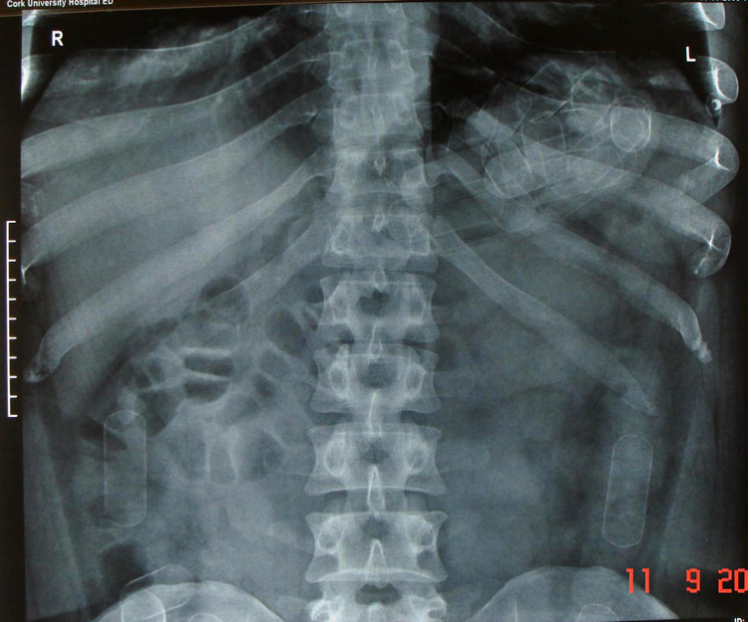
Plain radiograph of the abdomen demonstrated multiple homogenous radiopacities within the lumen of the bowel demonstrating the typical appearances of Type IV packages.

**Figure 2:**
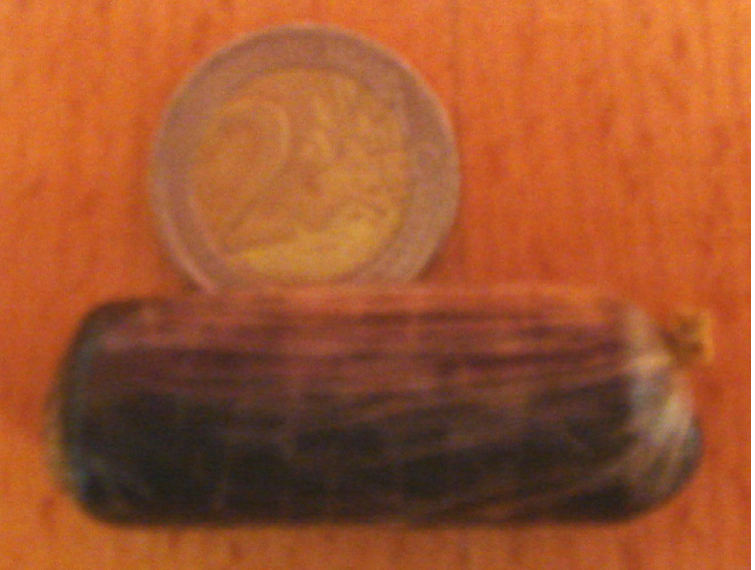
Photograph demonstrating thick wall of a capsule with white substance (found subsequently to be cocaine paste) within.

A 34 year old male was brought to the Emergency Department of our hospital by the local airport police authority having admitting swallowing 34 capsules of cocaine. On presentation the patient was asymptomatic, had normal vital signs and physical examination was unremarkable. His electrocardiogram was normal and blood testing including full blood count, renal indices and liver blood tests were all within normal limits. A plain film of the abdomen was performed and showed multiple homogenous, regularly shaped radiopacities within the lumen of the gastrointestinal tract consistent with packages (see Figure 1). The patient was therefore observed for passage of the capsules with defecation. Once passed per rectum the capsules were seen be encased in a dark, hard covering which required some considerable force to be opened in order to reveal the contents (see Figure 2) – these represent "Type IV packages" (see Table 1) [1]. He underwent monitoring with serial plain radiology in order to ensure complete transit of the capsules (34 in total). This took 96 hours during which time he remained entirely asymptomatic. He was subsequently discharged to the care of the police.

**Table 1:** Classification by category of packages used for ingestion by drug smugglers.

**Categorical types of packages.**	
Type I	Loosely packed cocaine covered by two to four layers of condoms or other latex-like material. This type has the highest risk for leakage/rupture.
Type II	Tightly packed cocaine powder or paste covered in multiple layers of tubular latex
Type III	Tightly packed cocaine powder or paste covered by aluminium foil.

*Types 1, 2 and 3 are radiolucent.*	

Type IV	Dense cocaine paste is placed into a device, condensed and hardened. This is then packaged in tough tubular latex. This is then covered with coloured paraffin or fibreglass. It is always radiopaque, rendering it easily indentifiable on plain X-ray of the abdomen.

## Discussion

Ingesting multiple packets of drugs ("body packing") for the purpose of evading police detection is a well-described method of smuggling although the actual frequency is unknown, as most will go undetected. The process carries risks other than criminal charges. Acute drug intoxication due to rupture of the package(s) within the gastrointestinal tract leads to inadvertent over-dosage as the packages contain concentrated cocaine and can be fatal. Unlike heroin toxicity, there is no direct antidote for cocaine toxicity and the mortality rate after package leakage approaches 60% [2].

While it is clear that small bowel obstruction is an obvious indication for surgical removal, the indications for premptive surgery to obviate the risk of rupture have changed in recent years as body packers have developed more sophisticated packaging methods. McCarron and Wood initially detailed the three different types of cocaine packaging to which has been added a fourth type by Pidoto et al. [1]. Therefore while initial recommendations strongly advocate early surgery once the diagnosis is apparent, these were based on experience with type I and II body packers with their high and unpredictable risk of package leakage/rupture. With the development of type III packets, a more conservative medical management was adopted and later publications counsel observation unless symptoms develop. The tough exterior of type VI packaging makes the risk of leakage/rupture very low and so its popularity with body packers has increased.

The standard examination for detection and surveillance is plain X-ray of the abdomen in an upright and a supine position. Depending on the purity of the drug, three different forms of attenuation have been described for types I-III: hashish is denser than stool; cocaine appears similar to stool; and heroin has a gaseous transparence. Computed tomography is occasionally used but nevertheless described as a very accurate diagnostic tool. Ultrasound and MR imaging do not play an important role. Regular urine analysis may reassure regarding the safety of this approach. Prolonged observation may be required as the packers often take bowel constipaters to avoid defecation at inopportune times during their journey.

**Figure 3:**
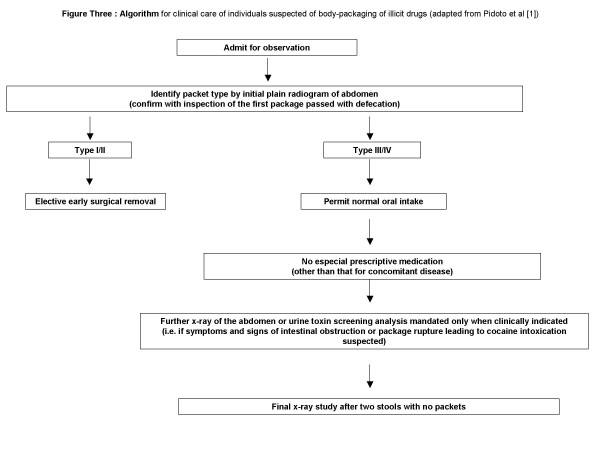
**Algorithm **for clinical care of individuals suspected of body-packaging of illicit drugs (adapted from Pidoto et al [1]).

When surgical extraction is necessary the focus must be on removing all packets. One surgical review of the topic recommends that a single enterotomy is usually sufficient to do this as the packages can usually be "milked" to the enterotomy site and evacuated manually [3]. The tough exterior coat on type VI packages facilitates their milking along the gastrointestinal tract without the risk of rupture seen with types I, II and III. Other reports however warn that wide dispersion of packets in the gut may make intraoperative detection difficult and suggests that multiple incisions may be required [4]. The most comprehensive modern series of type VI packers is that of Pidoto et al who reported on 161 cases of body packers over a two year period managed in their institution. Their surveillance protocol permitted only minimal medical intervention. In their experience, only five patients (3%) needed laparotomy. Three patients needed gastrotomy for symptoms of gastric occlusion while two were able to have the packages milked along their distal small bowel and colon so that expulsion of the packages could be achieved through the rectum thus avoiding the need for enterotomy. "Modern-day" body packagers without symptoms of bowel obstruction or overdosage may therefore be observed with confidence once the typical radiological appearances are present (see Figure 3 – algorithm for suggested care pathway).
